# NMR spectroscopic and analytical ultracentrifuge analysis of membrane protein detergent complexes

**DOI:** 10.1186/1472-6807-7-74

**Published:** 2007-11-08

**Authors:** Innokentiy Maslennikov, Georgia Kefala, Casey Johnson, Roland Riek, Senyon Choe, Witek Kwiatkowski

**Affiliations:** 1 Structural Biology Laboratory, The Salk Institute for Biological Studies, 10010 North Torrey Pines Rd., La Jolla, USA, CA 92037

## Abstract

**Background:**

Structural studies of integral membrane proteins (IMPs) are hampered by inherent difficulties in their heterologous expression and in the purification of solubilized protein-detergent complexes (PDCs). The choice and concentrations of detergents used in an IMP preparation play a critical role in protein homogeneity and are thus important for successful crystallization.

**Results:**

Seeking an effective and standardized means applicable to genomic approaches for the characterization of PDCs, we chose 1D-NMR spectroscopic analysis to monitor the detergent content throughout their purification: protein extraction, detergent exchange, and sample concentration. We demonstrate that a single NMR measurement combined with a SDS-PAGE of a detergent extracted sample provides a useful gauge of the detergent's extraction potential for a given protein. Furthermore, careful monitoring of the detergent content during the process of IMP production allows for a high level of reproducibility. We also show that in many cases a simple sedimentation velocity measurement provides sufficient data to estimate both the oligomeric state and the detergent-to-protein ratio in PDCs, as well as to evaluate the homogeneity of the samples prior to crystallization screening.

**Conclusion:**

The techniques presented here facilitate the screening and selection of the extraction detergent, as well as help to maintain reproducibility in the detergent exchange and PDC concentration procedures. Such reproducibility is particularly important for the optimization of initial crystallization conditions, for which multiple purifications are routinely required.

## Background

A bottleneck in x-ray crystallography-based structural genomics projects of integral membrane proteins (IMPs) is obtaining diffracting crystals of the IMPs. The difficulties associated with the purification and crystallization of these molecules have caused crystallographic studies of IMPs to lag 25 years behind those of water-soluble proteins [1,2]. Today, there are over 43,000 structure entries for water-soluble proteins deposited in the public Protein Data Bank [3,4], in contrast to fewer than 800 IMP entries (corresponding to less than 200 unique structures). This discrepancy is largely due to the fact that the crystal structure determination of an IMP is often experimentally hampered by difficulties associated with its heterologous expression, solubilization by detergents, purification, and crystallization [5,6]. Recent advances in high-throughput (HT) technologies enable us to partly overcome these difficulties through the application of genomic approaches. For instance, an efficient fusion system [7] allows the screening of a large number of related or homologous targets to find those that overexpress and behave well for further characterization. The characterization of IMP behavior in different detergents includes many important factors, such as functional activity, homogeneity and stability. While the characterization of a particular IMP's functional activity (often depending on the presence of natural or synthetic ligands or lipids) cannot be generalized, the quantification of the detergents and their effects on the homogeneity of the protein-detergent complex (PDC) can be standardized, and are thus the focus of this paper. Extensive characterization of the PDC while screening for suitable detergents is inevitably time-consuming. However, failing to monitor detergent exchange or to control the detergent-to-protein ratio in the course of membrane solubilization and protein purification often results in inconsistent sample preparations and uncertainty of the PDCs' structural homogeneity. Therefore, we sought an effective and relatively fast means to characterize PDCs, which could be applied in combination with genomic strategies.

We chose NMR spectroscopy to monitor the composition of detergents in the detergent-containing samples. NMR, unlike other methods for determining detergent concentration, such as refractive index [8], Fourier-transform infrared spectroscopy [9], and thin layer chromatography [10], offers the advantage of confirming the chemical identity unambiguously while simultaneously quantifying the components in the sample. Furthermore, establishing the oligomeric homogeneity of a PDC is an important step before carrying out extensive crystallization screening of the target protein [11-13]. There are several analytical techniques that can be used to measure sample homogeneity, such as analytical ultracentrifugation [14-16] and static light scattering/refractive index measurements coupled with size exclusion chromatography [8,17]. Here, we used the traditional analytical ultracentrifugation method, adapting it to our purpose of monitoring sample homogeneity while simultaneously providing a rough estimate of the oligomeric state and the detergent-to-protein ratio of a sample in a quick and simple experiment.

By applying these two analytical methods, it is possible to quickly establish the optimum detergent concentration during IMP extraction from the membrane, to control its subsequent exchange into alternate detergents, to monitor the detergent content during the processes of purification and concentration of the protein sample, and to evaluate its oligomeric homogeneity. We find that the ability of a detergent to solubilize an IMP is dependent not only on protein identity but also on its expression level.

## Results and Discussion

### Step 1: Protein extraction from cell membranes

Following protein expression in *Escherichia coli *(*E. coli*), a crude membrane fraction (for preparation details, see Methods) containing an overexpressed IMP is solubilized by a detergent of choice as the first step of IMP purification. To screen for the optimal detergent for protein extraction from the cell membrane, we used nine detergents belonging to three different classes: non-ionic, zwitterionic and anionic (initial concentrations in extraction buffer are shown in Table 1). These detergents were used individually to extract the test protein, QseC, a histidine kinase receptor from *E. coli*, by incubating the crude membrane fraction overnight in a detergent-containing buffer. Attempting to ensure sufficient solubilizing power of the extraction buffer, we used a detergent concentration of at least 5 times the critical micelle concentration (CMC) or 0.1 mM micellar concentration, whichever was higher. The micellar concentration is defined here as the molar concentration of the detergent divided by its aggregation number. The detergent-solubilized fraction was then separated from insoluble material by centrifugation at 125,000 g (high-speed spin), the supernatant of which was then analyzed by SDS-PAGE (see Methods for details).

**Table 1:** General properties of detergents used.

Detergent	MW of monomer [Da]	cMc [mM] ^a^	Aggregation number ^a^	Theoretical v¯d MathType@MTEF@5@5@+=feaafiart1ev1aaatCvAUfKttLearuWrP9MDH5MBPbIqV92AaeXatLxBI9gBaebbnrfifHhDYfgasaacPC6xNi=xH8viVGI8Gi=hEeeu0xXdbba9frFj0xb9qqpG0dXdb9aspeI8k8fiI+fsY=rqGqVepae9pg0db9vqaiVgFr0xfr=xfr=xc9adbaqaaeGacaGaaiaabeqaaeqabiWaaaGcbaGafmODayNbaebadaWgaaWcbaGaemizaqgabeaaaaa@2EDD@^b^	Concentration used in extraction buffer [mM]	References
*Zwitterionic*						
**Zw-3.14 **^c^	**363.6**	**0.16**	**83–130 (130 **^d^)	**0.9714**	**14.5**	[24, 25]
**Zw-3.12**	**335.0**	**2.8 **^e^	**55–87 (80 **^d^)	**0.9568**	**15.3**	[25, 26]
**LDAO**	**229.4**	**1–2**	**76**	**1.0597**	**16.0**	[27]
**FC14**	**379.5**	**0.12**	**80–120 **^f ^**(100 **^d^)	**0.9876**	**5.3**	[8, 24]
FC12, DPC	351.5	1.5	50–60	0.9747	-	[24, 25]
*Anionic*						
**SDS**	**288.4**	**2.6 **^g^	**62–101 (100 **^d^)	**0.8578**	**17.5**	[24, 28]
DPh	373.4			0.8123	-	[24]
*Non-ionic*						
**Brij-35**	**1198 av.**	**0.091**	**40**		**4.3**	[25, 29]
**C12E8**	**538.8**	**0.09 **^g^	**90–120 **^h ^**(100 **^d^)		**9.6**	[25]
**Triton X100**	**647 av.**	**0.23**	**75–165 (160 **^d^)		**16.0**	[25, 30]
**DM**	**482.6**	**1.8**	**69**	**0.7725**	**12.0**	[24, 31]
DDM	510.6	0.17	78–149	0.7932	-	[24, 28]
NG	306.4	6.5		0.8495	-	[24]
OG	292.4	18–20	27–100	0.8352	-	[32]

^a ^– in H_2_O, if otherwise is not specified; ^b ^– from [18] or calculated according to [18]; ^c ^– detergents used for extraction screen are in bold font; ^d ^– averaged values used in calculations; ^e ^– 20 mM Tris-HCl, pH 8.0, 100 mM NaCl; ^f ^– estimated value; ^g ^– pH 7.5; ^h ^– 10 mM TES, pH 7.5, 50 mM NaCl, 0.1 mM CaCl_2_

We measured the actual concentration of the detergent in the starting extraction buffer as well as in the detergent-solubilized fraction by NMR spectroscopy in order to calculate a detergent retention ratio. The ratio is defined as the concentration of the detergent in the high speed spin supernatant fraction to that in the initial extraction buffer. To derive the accurate quantity of the detergent, we used the integral intensity of signature ^1^H-signals in NMR spectra established for the ten detergents in this study (Table 2) calibrated against a standard (2,2-dimethyl-2-silapentane-5-sulfonic acid, DSS) at a known concentration. Results were crosschecked by measuring integrals of the several ^1^H-signals of the detergent (see Methods for details). The detergent molar concentration in PDC samples is usually at least 50 times the protein molar concentration. Hence the impact of the overlapping protein signals on calculated detergent concentration is within the margin of experimental error (baseline correction and peak integration), estimated to be less than 5%.

**Table 2:** Characteristic ^1^H signals of used detergents.

Detergent	Group	Chemical shift [ppm]	Number of protons	Minimal detectable concentration [×10^-3 ^mM]^a^
FC14, FC12	(CH_3_)_3_-N-	3.23	9	10
	CH_3_-	0.86	3	30
DDM, DM	-C^1^H-	4.45	1	200^b^
	-C^1^H-	5.34	1	200^b^
	CH_3_-	0.86	3	30
DPh	CH_3_-	0.86	3	30
	-N-((CH_2_)_2_-COO^-^)_2_	2.4–3.1^c^	8	30
SDS	CH_3_-	0.86	3	30
	-CH_2_-(SO_4_)^-^	4.02	2	50
NG, OG	-C^1^H-	4.45	1	200^b^
	CH_3_-	0.86	3	30
Zw-3.14, LDAO	-(CH_3_)_2_	3.11	6	15
	CH_3_-	0.86	3	30
DSS (reference)	(CH_3_)_3_-Si-	0.00	9	

^a ^– approximate lower limit of measurable concentration for the samples in 10–20% D_2_O, measured using Varian NMR System 500 MHz, probe sensitivity 1:350, 64 scans.

^b ^– 100 × 10^-3 ^mM in 100% D_2_O.

^c ^– the chemical shifts of these signals are pH- and concentration-dependent.

**Figure 1:**
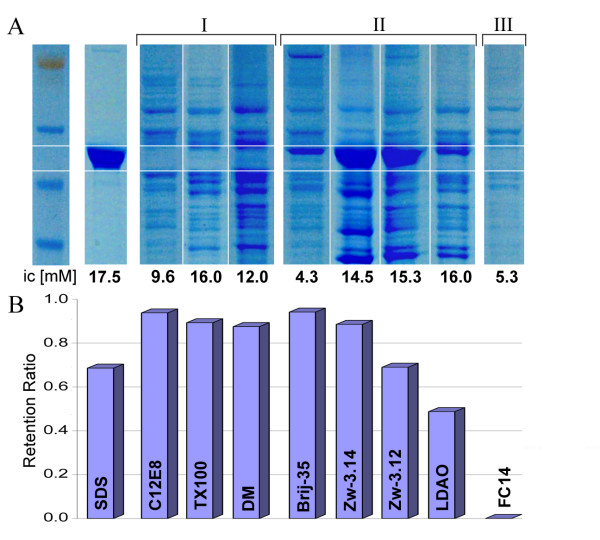
**Detergent extraction screen**. (A) NuPAGE (4–12%) Bis-Tris SDS gel of the high-speed spin (125,000 g, 2 h) supernatant of the membrane fraction of QseC, extracted overnight by different detergents. Each well was loaded with 10 *μ*l of a 1:1 supernatant-to-loading buffer solution, except for the SDS sample, for which 2 *μ*l was loaded to adjust for a comparable amount of protein in the gel. (B) The detergent retention ratio defined as *fc*/*ic *is shown for each detergent of panel A (*ic *is the initial detergent concentration, *fc *is the final detergent concentration, both determined by NMR).

Figure 1 shows that different detergents display widely varying degrees of extraction efficiency. Here, QseC extracted by SDS, a denaturing ionic detergent, provides a reference point as the maximum quantity of QseC extractable from the cell membrane. The detergents used in the extraction experiment can be divided into 3 general groups with respect to their QseC extraction efficiency and retention ratio at the concentrations used. Three detergents with a high retention ratio do not extract QseC (Figure 1, Group I), while four others that have retention ratios between 0.5 and 1 extract QseC with varying degrees of efficiency (Figure 1, Group II). A single detergent tested, FC14, gave a retention ratio of nearly 0 and did not extract QseC (Figure 1, Group III). Measurements of detergent concentration by NMR indicate that after the overnight incubation some or all of the detergent in Groups II and III (e.g. 50% of LDAO or >99% of FC14) were pelleted by the high-speed spin. The disappearance of the detergent ^1^H-signals cannot be attributed to line broadening in large soluble PDC aggregates, because even in PDCs both the hydrophobic chain and the hydrophilic headgroup of a detergent are mobile enough to produce ^1^H-signals of sufficiently narrow linewidth (within the ranges of 6–20 Hz for protons of hydrophobic chains and 3–6 Hz for protons of headgroups at 500 MHz ^1^H frequency). Furthermore, since no QseC is detected by SDS-PAGE in the FC14-solubilized fraction, for which the retention ratio is nearly 0, the only plausible explanation is that the detergent is in the pellet.

In order to determine if the depletion of a detergent is due to binding with QseC itself, which is present in large quantities in the crude membrane fraction, or to the natural lipid membrane of *E. coli*, we performed control measurements on the membrane extract from the same amount of uninduced cells. In the absence of an overexpressed IMP, none of the detergents was significantly depleted by high-speed centrifugation. This clearly indicates that detergent depletion is primarily due to the presence of large amounts of QseC expressed in the membrane rather than an inability to completely solubilize the lipid membrane. Thus it seems most reasonable to surmise that FC14 did bind to QseC but that the protein remained insoluble and that these PDC aggregates were pelleted by the high-speed spin (Figure 1).

**Figure 2:**
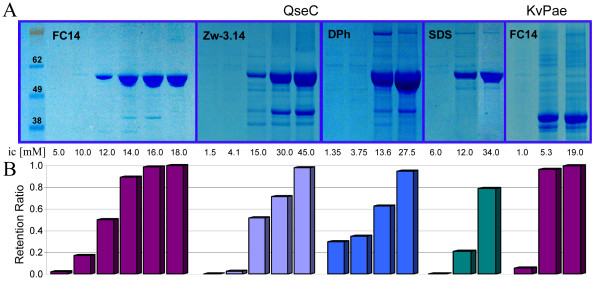
**Detergent concentration screen**. (A) NuPAGE (4–12%) Bis-Tris SDS gel of the high speed spin (125,000 g, 2 h) supernatant from the overnight extraction of the membrane fraction. Extraction detergents FC14, Zw-3.14, DPh, and SDS with increasing concentrations were tested on QseC and KvPae. Sample loading is as in 1A. (B) The detergent retention ratios for each extraction condition.

Since the depletion of FC14 by QseC was particularly pronounced, we increased the concentration of FC14 incrementally to test if there was a threshold concentration that would be sufficient to extract QseC. In the same manner we also tested a Group II detergent – Zw-3.14 – and two efficient extractors – SDS and DPh. Figure 2 shows that the increasing amount of QseC extracted by FC14 is strongly correlated with the increasing detergent retention ratio. The same threshold behavior is also evident for Zw-3.14, DPh, and SDS, as well as for FC14 when it was used to extract another IMP (the K^+ ^Channel-like protein from *Pseudomonas aeruginosa*, KvPae). KvPae was fully extractable by FC14 (Figure 2), however the maximum extraction efficiency was reached at a lower concentration, displaying a threshold concentration approximately 4–5-times lower than that observed for QseC. This correlates well with the expression level of KvPae, which was roughly 5 times lower than that of QseC.

These data furnish an important guideline for choosing a detergent and its concentration for protein extraction from the membrane. In the given example, Group I detergents are inadequate for QseC extraction from the membrane despite their high retention ratios (>0.9). On the other hand, the concentration of detergents that either display some extraction potential by SDS-PAGE or have very low retention ratios (Group II and III) must be adjusted so that their retention ratio is ~0.9 (requiring an NMR measurement of the detergent concentration in the solubilized fraction). Using these guidelines, we have overexpressed every *E.coli *receptor-kinase, adjusting the concentration of the detergent for the most efficient extraction (data not shown). For other classes of IMPs one must first establish the detergent extraction profile and then adjust the concentration of the detergent to achieve a retention ratio close to 0.9.

We showed that the threshold and optimum extraction concentration of a detergent depends on the identity and expression level of the protein. In the course of membrane solubilization, detergents compete against lipid molecules in the membrane to encircle IMP molecules. Detergents with a high specific affinity for the protein will have preferential binding to the protein embedded in the membrane, destabilizing the protein-lipid interaction. However, to break the protein-lipid contacts and solubilize an IMP, the ratio of detergent to protein has to exceed a certain threshold. This threshold depends not only on the affinity of the detergent to a given protein but also on the concentration of the protein in the membrane. The ability to easily measure the optimal concentration of detergent by NMR for a given system allows for an efficient IMP extraction using a minimum amount of what is often an expensive detergent.

### Step 2: Detergent exchange

**Figure 3:**
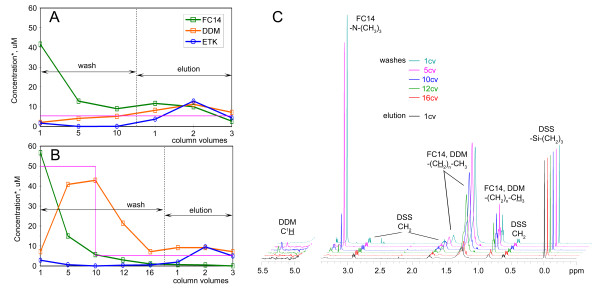
**Protein concentrations and micellar concentrations of extraction and destination detergents during detergent exchange on the Ni-NTA column**. Incomplete (A) and successful (B) exchange of FC14 with DDM. Detergent micellar concentrations are shown in green (FC14) and orange (DDM); protein concentrations are shown in blue; the applied micellar concentration of DDM is shown in magenta. (C) ^1^H-NMR spectra (5.5...4.9 ppm and 3.4...-0.5 ppm regions) of the wash and elution fractions in B, collected during the exchange of FC14 with DDM on a Ni-NTA column: cyan – 1 column volume (cv) wash; magenta – 5 cv wash; blue – 10 cv wash; green – 12 cv wash; red – 16 cv wash and black – 1 cv elution. The spectra are normalized according to the integral intensity of the DSS (CH_3_)_3_-Si-group signal (0.0 ppm) and stacked for comparison. The FC14 concentration was calculated based on the integral of the (CH_3_)_3_-N- signal at 3.23 ppm. The DDM concentration was calculated based on the integral of the C^1^H signal at 5.34 ppm for washes 3, 5 and 6 and the integral of -(CH_2_)_n_-CH_3 _peak for wash 8 and elution 1, to which contribution from FC14 was known based on the integral of the (CH_3_)_3_-N-signal.

During the course of an IMP preparation, it is often necessary to change the detergent of the PDC. The question of how efficient it is to exchange one detergent for another, especially for a less hydrophobic one, is often left unasked, yielding mixed results at the detergent exchange step. To answer this question, we carried out a detergent exchange of FC14, which was originally used to extract Etk (an *E. coli *receptor tyrosine kinase), for less hydrophobic detergents: DDM, DM, NG and OG, while monitoring detergent concentrations in all samples. Because the recombinant Etk contains an octa-histidine tag, we performed detergent exchange while Etk, extracted by 18 mM of FC14, was immobilized on a Ni-NTA column and washed with several column volumes of the destination detergent followed by elution with 250 mM imidazole in the destination detergent. We monitored the concentrations of the extraction and destination detergents, as well as total protein concentration in the flow-through, wash, and elution fractions from the Ni-NTA column. Micellar concentrations of FC14 and DDM are plotted together with protein concentration for each fraction in Figure 3A and 3B. Figure 3A shows that FC14 is not efficiently replaced by DDM even after washing with 10 column volumes of 0.5 mM DDM. The second of the three 1-column volume elution fractions has a micellar concentration of FC14 that is still equal to the protein concentration (Figure 3A), indicating an unsuccessful exchange of FC14 for DDM. However, when the concentration of DDM was increased to 5 mM during the wash, complete exchange of FC14 was achieved (Figure 3B). Before elution, the concentration of the destination detergent was reduced by additional washes (6-column volumes) containing 0.5 mM DDM. The complete exchange was confirmed by the integrals of the signature peaks of FC14 and DDM (Table 2, Figure 3C). The destination detergent DM also successfully replaced FC14 only when used at a higher concentration during the wash step. Attempts to replace FC14 with the even less hydrophobic detergents, NG and OG, caused protein precipitation. These results are the first quantitative data on detergent exchange that demonstrate the importance of an extensive wash with a high concentration of the destination detergent.

### Step 3: Concentrating protein-detergent complexes (PDCs)

Protein concentrations on the order of 0.5 mM are often employed for growing crystals and the most common technique for attaining reproducible protein concentrations is ultrafiltration. However, this technique often results in an unpredictable, concomitant increase in the protein-free micelle concentration, making protein crystallization less reproducible from preparation to preparation. Some details of detergent behavior during IMP concentration were recently reported [8] for four popular detergents using Amicon ultrafiltration devices with 30 kDa and 100 kDa molecular weight cutoff (MWCO) membranes. We measured detergent concentrations in retained and flow-through fractions for ten detergents commonly used for protein purification and crystallization (see Methods). We compared results from concentrating these detergents in Amicon and Vivaspin ultrafiltration devices with 30 and 50 kDa MWCOs. The initial detergent concentrations were set to levels commonly used in IMP purification. All detergents are concentrated to some extent in both the Amicon and Vivaspin 30 kDa cutoff concentrators (Table 3), and, as expected, detergents that form larger micelles tend to be concentrated more than those forming smaller micelles.

**Table 3:** Detergent concentration factors *cf *(see Methods) for the Amicon and Vivaspin concentrators with 30 and 50 KDa cutoffs for several detergents, using initial concentrations *ic*, and fold concentrations set as 4× for Amicon and 5× for Vivaspin, unless marked otherwise.

		Amicon			Vivaspin		
		
			*cf*			*cf*	
					
Detergent	MW micelle [kDa]	*ic *[mM]	30 kDa	50 kDa	*ic *[mM]	30 kDa	50 kDa
FC14	46 ^a^	0.34 ^b^	0.48	0.30	0.34 ^b^	0.39	0.03
		0.55	0.79	0.73	0.43	0.48	0.03
DDM	40–76 ^c, d^	0.35 ^b^	0.31	0.23	0.35 ^b^	0.17	0.00
		0.54	0.64	0.56	0.54	0.57	0.00
Zw-3.14	30–47 ^e^	1.04	0.62	0.40	1.17	0.31	0.21
FC12	18–21 ^c^	3.60 ^b^	0.30	0.21	3.60 ^b^	0.05	0.01
		9.20 ^b^	0.52	0.23	9.20 ^b^	0.24	0.02
DM	33.3 ^c^	7.22	0.55	0.46	8.33	0.62	0.01
SDS	18–29 ^e^	9.21	0.33	0.28	10.00	0.71	0.31
DPh	20–30 ^f^	9.28	0.31	0.29	9.87	0.42	0.07
LDAO	17 ^g^	0.51 ^b^	0.01	0.01	0.51 ^b^	0.00	0.00
		2.18 ^b^	0.32	0.27	2.18 ^b^	0.15	0.01
		5.10 ^b^	0.50	0.32	5.10 ^b^	0.28	0.04
		10.28	0.68	0.64	10.28	0.75	0.20
NG	15–30 ^f^	16.50	0.13	0.01	18.93	0.39	0.03
OG	8–26 ^e, h^	38.30	0.05	0.00	44.17	0.21	0.03

^a ^– [8]; ^b ^– the sample was concentrated 10×; ^c ^– [24]; ^d ^– [28]; ^e ^– [25]; ^f ^– our estimates; ^g ^– [27]; ^h ^– [32].

**Figure 4:**
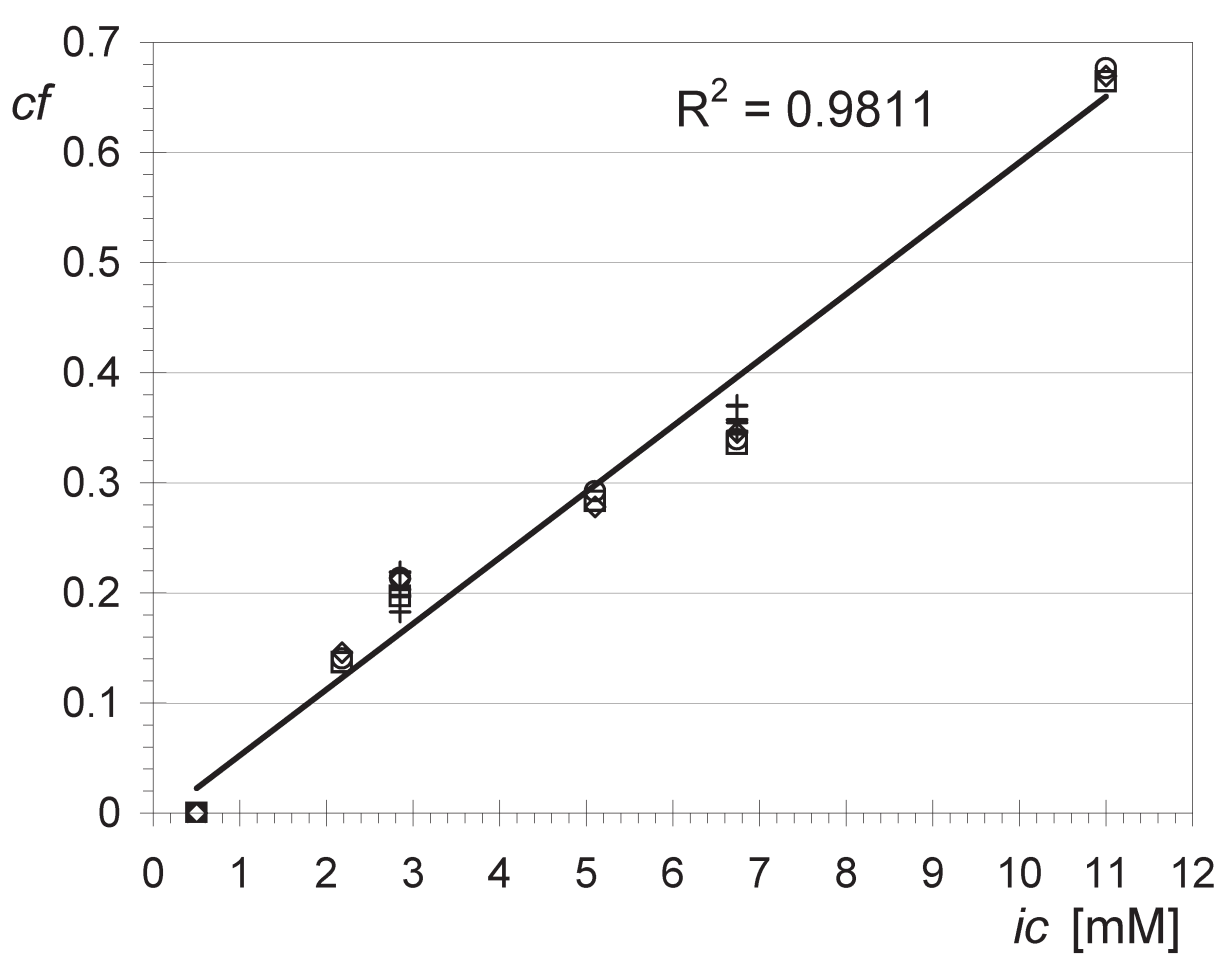
**Dependence of the detergent concentration factor on the initial detergent concentration**. Dependence of the concentration factor (see Methods) on the initial LDAO concentration. Detergent samples were prepared in 20 mM Tris-HCl buffer, pH 8.0 and concentrated 10× using Vivaspin 30 KDa cutoff ultrafiltration devices. Concentration factors were calculated using concentration measurements based on three different peaks in ^1^H-NMR spectra (at 3.11, 1.27 and 0.86 ppm). For the initial concentrations of 2.8 and 6.8 mM, three different devices were used and the concentration factors were calculated for each sample (additional data points are shown as "+"). The least-squares regression line and the corresponding R^2 ^are shown.

Ideally, the concentration factor (0 = no retention, 1 = all retained; see Methods) should be independent of the starting concentration and the concentration fold, however, the process of filtering detergents is more complex. To address the discrepancy between our data and the data from [8], we checked the effect of the starting concentration on the observed concentration factor for LDAO. From the plot in Figure 4 we conclude that the concentration factor for LDAO increases linearly with its initial concentration. However, even for the same ultrafiltration device (30 kDa Amicon), at the same starting concentration (*ic *= 2.18 mM), and with the same fold concentration (n = 10) for LDAO, there is still a significant difference between the concentration factors calculated from our data (*cf *= 0.32) and the data published in [8] (*cf *= 0). To eliminate the possibility of an effect due to device to device variability we concentrated LDAO using three devices of the same kind at two initial concentrations (2.8 and 6.8 mM). We observe a variability of less than 20% within devices of the same manufacturer and membrane cutoff. Therefore, it is likely that different factors such as LDAO purity, temperature, or pH are at play.

It is not clear what causes the unexpected increase in the concentration factor with increasing initial concentration, but one possibility is the concentration-dependent equilibrium between micelles and detergent monomers.

Nevertheless, the result stresses the importance of monitoring the detergent concentration in order to obtain reproducible PDC preparations, and that even the initial concentration of detergent and the fold concentration can impact the final detergent concentration. Another important factor to keep in mind is the differences between the models of ultrafiltration devices. A comparison of the 50 kDa MWCO Vivaspin and Amicon devices revealed an interesting discrepancy. The Vivaspin PES membrane is permeable for all but three tested detergents, however, the corresponding Amicon device (regenerated cellulose membrane) is only permeable to NG and OG (Table 3), as if the 50 kDa Amicon membrane acted as a filter with the cutoff size for detergent micelles smaller than specified.

### Step 4: Analysis of the homogeneity of the PDCs by analytical ultracentrifugation

The Tanford and Reynolds method was established to study PDCs by analytical ultracentrifugation [14]. The core of the method relies on adjusting the density of the solvent to eliminate the detergent's contribution to the buoyant mass of the PDC. Specifically, the Tanford equation states that:

*M*_*b *_= *M*_*p*_·(1 - *ρ*·v¯p
 MathType@MTEF@5@5@+=feaafiart1ev1aaatCvAUfKttLearuWrP9MDH5MBPbIqV92AaeXatLxBI9gBaebbnrfifHhDYfgasaacPC6xNi=xH8viVGI8Gi=hEeeu0xXdbba9frFj0xb9qqpG0dXdb9aspeI8k8fiI+fsY=rqGqVepae9pg0db9vqaiVgFr0xfr=xfr=xc9adbaqaaeGacaGaaiaabeqaaeqabiWaaaGcbaGafmODayNbaebadaWgaaWcbaGaemiCaahabeaaaaa@2EF5@) + *M*_*d*_·(1 - *ρ*·v¯d
 MathType@MTEF@5@5@+=feaafiart1ev1aaatCvAUfKttLearuWrP9MDH5MBPbIqV92AaeXatLxBI9gBaebbnrfifHhDYfgasaacPC6xNi=xH8viVGI8Gi=hEeeu0xXdbba9frFj0xb9qqpG0dXdb9aspeI8k8fiI+fsY=rqGqVepae9pg0db9vqaiVgFr0xfr=xfr=xc9adbaqaaeGacaGaaiaabeqaaeqabiWaaaGcbaGafmODayNbaebadaWgaaWcbaGaemizaqgabeaaaaa@2EDD@),

where *M*_*b *_is the buoyant mass of the PDC, *M*_*p *_and *M*_*d *_are the protein and detergent masses, v¯p
 MathType@MTEF@5@5@+=feaafiart1ev1aaatCvAUfKttLearuWrP9MDH5MBPbIqV92AaeXatLxBI9gBaebbnrfifHhDYfgasaacPC6xNi=xH8viVGI8Gi=hEeeu0xXdbba9frFj0xb9qqpG0dXdb9aspeI8k8fiI+fsY=rqGqVepae9pg0db9vqaiVgFr0xfr=xfr=xc9adbaqaaeGacaGaaiaabeqaaeqabiWaaaGcbaGafmODayNbaebadaWgaaWcbaGaemiCaahabeaaaaa@2EF5@ and v¯d
 MathType@MTEF@5@5@+=feaafiart1ev1aaatCvAUfKttLearuWrP9MDH5MBPbIqV92AaeXatLxBI9gBaebbnrfifHhDYfgasaacPC6xNi=xH8viVGI8Gi=hEeeu0xXdbba9frFj0xb9qqpG0dXdb9aspeI8k8fiI+fsY=rqGqVepae9pg0db9vqaiVgFr0xfr=xfr=xc9adbaqaaeGacaGaaiaabeqaaeqabiWaaaGcbaGafmODayNbaebadaWgaaWcbaGaemizaqgabeaaaaa@2EDD@ are the partial specific volumes of protein and detergent, and *ρ *is the measured density of the sample buffer. Buffer density can be adjusted by using mixtures of H_2_O and D_2_O so that the second term of the equation containing (1 - *ρ*·v¯d
 MathType@MTEF@5@5@+=feaafiart1ev1aaatCvAUfKttLearuWrP9MDH5MBPbIqV92AaeXatLxBI9gBaebbnrfifHhDYfgasaacPC6xNi=xH8viVGI8Gi=hEeeu0xXdbba9frFj0xb9qqpG0dXdb9aspeI8k8fiI+fsY=rqGqVepae9pg0db9vqaiVgFr0xfr=xfr=xc9adbaqaaeGacaGaaiaabeqaaeqabiWaaaGcbaGafmODayNbaebadaWgaaWcbaGaemizaqgabeaaaaa@2EDD@) becomes 0, thus eliminating the detergent contribution to the equation and enabling direct measurement of protein mass in the PDC. This works well for popular detergents such as FC14 or FC12, but the densities of many other popular detergents including DM or DDM are outside the adjustable range: from 1.0 g/ml (100% H_2_O) to 1.1 g/ml (100% D_2_O). To overcome this limit, Tanford measured *Mb *in a series of H_2_O/D_2_O mixtures, which not only allowed him to extrapolate *M*_*b *_at the density point at which (1 - *ρ*·v¯d
 MathType@MTEF@5@5@+=feaafiart1ev1aaatCvAUfKttLearuWrP9MDH5MBPbIqV92AaeXatLxBI9gBaebbnrfifHhDYfgasaacPC6xNi=xH8viVGI8Gi=hEeeu0xXdbba9frFj0xb9qqpG0dXdb9aspeI8k8fiI+fsY=rqGqVepae9pg0db9vqaiVgFr0xfr=xfr=xc9adbaqaaeGacaGaaiaabeqaaeqabiWaaaGcbaGafmODayNbaebadaWgaaWcbaGaemizaqgabeaaaaa@2EDD@) = 0, and thus derive *M*_*p*_, but also to calculate the *M*_*d*_/*M*_*p *_ratio in the PDC [15]. A modification of this method using global nonlinear fitting instead of extrapolation that reduces the errors arising from extrapolation has been described [16]. However, these experiments remain too elaborate to be performed routinely and will bear significant experimental error for detergents, for which the extrapolation point is far from experimental data. For such detergents we propose a crude method of evaluating oligomeric state by introducing additional physical restraints to the equation so that a single velocity measurement yields a rough estimate of both the oligomeric state of the protein and the detergent content in the PDC. Since the buffer density *ρ *can be experimentally measured, and v¯p
 MathType@MTEF@5@5@+=feaafiart1ev1aaatCvAUfKttLearuWrP9MDH5MBPbIqV92AaeXatLxBI9gBaebbnrfifHhDYfgasaacPC6xNi=xH8viVGI8Gi=hEeeu0xXdbba9frFj0xb9qqpG0dXdb9aspeI8k8fiI+fsY=rqGqVepae9pg0db9vqaiVgFr0xfr=xfr=xc9adbaqaaeGacaGaaiaabeqaaeqabiWaaaGcbaGafmODayNbaebadaWgaaWcbaGaemiCaahabeaaaaa@2EF5@ and v¯d
 MathType@MTEF@5@5@+=feaafiart1ev1aaatCvAUfKttLearuWrP9MDH5MBPbIqV92AaeXatLxBI9gBaebbnrfifHhDYfgasaacPC6xNi=xH8viVGI8Gi=hEeeu0xXdbba9frFj0xb9qqpG0dXdb9aspeI8k8fiI+fsY=rqGqVepae9pg0db9vqaiVgFr0xfr=xfr=xc9adbaqaaeGacaGaaiaabeqaaeqabiWaaaGcbaGafmODayNbaebadaWgaaWcbaGaemizaqgabeaaaaa@2EDD@ can be calculated [18], the only variables to fit are *M*_*p *_and *M*_*d*_. If *M*_*p *_is reduced to *n*•*M*_*pm*_, where *M*_*pm *_is the mass of the monomeric protein, then the Tanford equation renders a set of discrete solutions {*n*, *M*_*dn*_} for *n *= 1,2,3... with a corresponding set of detergent masses, *M*_*dn*_. To select a plausible solution out of this set, an additional constraint is applied: since the detergent mass must be positive, the detergent-to-protein ratio (*M*_*d*_/*M*_*p*_) should be greater than 0 and, based on many experimental observations, less than 2.0 [19]. Additionally, with the same data, the homogeneity of the sample can be estimated by the van Holde-Weischet method [20].

**Figure 5:**
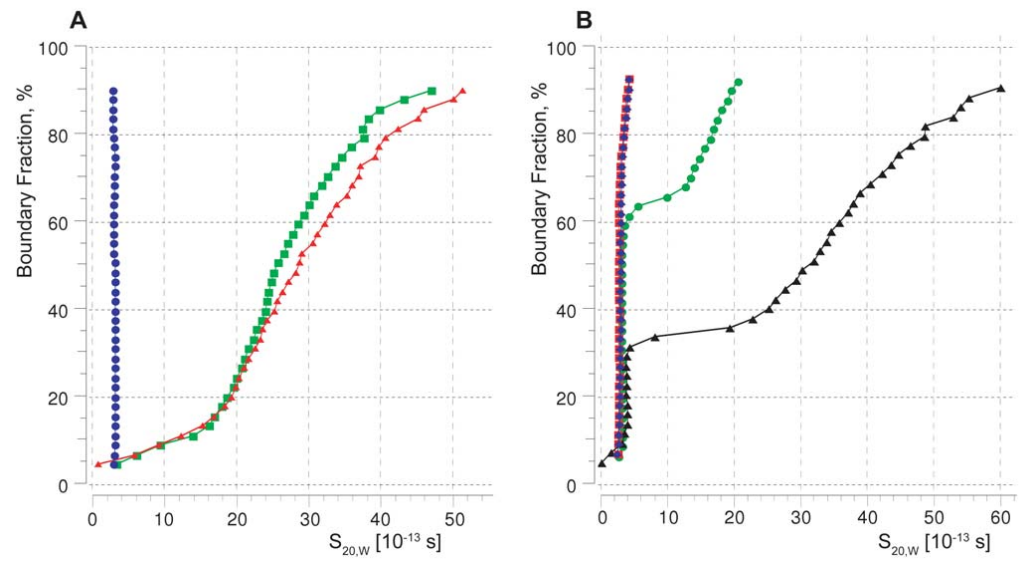
**Sedimentation velocity run analysis**. Distribution of the corrected (water, 20°C) sedimentation coefficient calculated using enhanced van Holde-Weischet analysis. Panel A: distribution analysis for EnvZ in FC14 (red line), in DDM exchanged from FC14 (green line) and in DDM exchanged from DPh (blue line). Panel B: distribution analysis for Etk in DDM exchanged from FC14 (black line), in FC12 (green line), and Etk-NM in FC12 (red line – with density adjustment; blue line – without density adjustment).

To illustrate these methods we performed velocity sedimentation [21] measurements on three constructs of two *E. coli *kinases after exchanging their extraction detergents for three popular detergents (DDM, FC14 and FC12) and taking a simple measurement of the density of the sample (see Methods). The velocity curves obtained for a histidine kinase receptor, EnvZ, and a tyrosine kinase receptor, Etk expressed as a Mistic-fusion protein [22] and Etk-NM (protein expressed without Mistic [22]), were analyzed using the ultrascan software package [23] and results are presented in Table 4. We can conclude from the results that despite the successful exchange of FC14 for DDM, EnvZ is highly aggregated (Figure 5A) and thus unsuitable for crystallization screening. This is also the case with EnvZ solubilized in its extraction detergent, FC14 (Figure 5A). However, EnvZ in DDM exchanged from DPh appears mostly homogeneous and most likely monomeric (Figure 5A, Table 4) and therefore suitable for crystallization trials. Thus, this simple measurement quickly demonstrates a critical difference between two extraction detergents that are both able to efficiently extract EnvZ from the membrane. The Etk sample in DDM is most likely a mixture of the low-oligomeric-weight protein in PDC and some higher-order aggregates (Figure 5B, Table 4). Exchanging extraction detergent for this protein with FC12 improved the ratio of the low-oligomeric-weight protein to the aggregates (Figure 5B) and increased stability of this protein. This sample was further purified by size exclusion chromatography and has yielded lead conditions from crystallization screening.

**Table 4:** Estimated oligomeric state of EnvZ in DDM exchanged from both FC14 and DPh, EnvZ in FC14, Etk in DDM exchanged from FC14, and Etk-NM in FC12. The solution of Tanford's equation for EnvZ in DDM, which satisfied the physical constraints (see Step 4), is highlighted.

		Detergent				
					
Protein	*M*_*pm *_[kDa]	Extraction	Exchange	Fitted mass [kDa]	*n*	*M*_*d*_*/M*_*p*_
EnvZ	65.206	FC14	FC14	Aggregates		
			DDM	Aggregates		
		DPh	DDM	112.2	***1***	***0.93***
					2	-0.18
Etk	99.788	FC14	DDM	~70% aggregates		
			FC12	~40% aggregates		
Etk-NM	84.737	FC12	FC12	83.3	1	
			FC12*	80.2	1	

* Density of the sample was adjusted to the density of FC12 (1.026 g/cm^3^)

On the other hand, the analysis of Etk-NM in FC12 showed predominantly monomeric protein (Figure 5B, Table 4). Homogeneity of the sample was confirmed using HPLC sizing chromatography, which displayed only one homogenous peak (data not shown). To assess the consistency of the method and the necessity of the density adjustment by D_2_O for FC12 we collected data for two samples of Etk-NM with and without adjustment. We found that the error of estimating mass for the unadjusted sample is less than 5% (Table 4), which is acceptable for a crude estimation of the oligomeric state and saves time in experiment preparation.

## Conclusion

The importance of monitoring the detergent content and homogeneity of the PDC during IMP preparation is illustrated by the behavior of detergents during extraction (Figure 1 and 2) and detergent exchange (Figure 3). Surprisingly, concentration efficiency depends not only on the initial concentration of the detergent (Figure 4), but also on the type of ultrafiltration membrane (Table 3). It is generally accepted that structural homogeneity of a protein sample should be achieved before dedicating time to screening crystallization conditions and the velocity sedimentation provides a quick method to monitor sample homogeneity (Figure 5).

The development of HT technology in the era of genomics has yielded an enormous increase in speed and efficiency that arises from advances in automation, miniaturization of traditional technology and adaptation of new technologies. While cloning and expression strategies have been developed for many protein production platforms, the purification of proteins has not benefited equally from automation, mostly due to the uniqueness of each protein. IMP purification adds yet another level of complexity in HT approaches. To standardize IMP production, there is a need for sensitive, rapid assays to characterize the detergent content and homogeneity of PDCs. We demonstrate that simple ^1^H-NMR spectroscopic analyses of PDCs provide a sound basis to standardize the steps of protein extraction, detergent exchange, and PDC concentration. We also illustrate that single velocity sedimentation measurements in H_2_O using common sense physical constraints on the model provide sufficient data to roughly estimate the oligomeric state and detergent-to-protein ratio in PDCs, and to evaluate the size homogeneity of the sample prior to crystallization screening. These methods are available to many labs and can be streamlined since dozens of samples can be measured overnight on an NMR spectrometer equipped with a sample exchanger and up to 21 samples a day can be measured on a single analytical ultracentrifuge instrument.

## Methods

### Materials

All detergents were purchased from Anatrace. NaCl, MgCl_2_, Trisma base, EDTA, Glycerol, BME and PMSF are from Sigma. EDTA-free "protease inhibitor cocktail" is from Roche. Vivaspin and Amicon concentrators are from Vivascience and Millipore, respectively.

### Membrane preparation

The genes were cloned to the Gateway-adapted pMIS vector [7] and/or pHIS (pMIS without Mistic) [22] and expressed in *E. coli *BL21 DE3 cells. Cells were grown overnight at 18°C after induction by 0.5 mM IPTG at OD_600 _= 1, harvested, resuspended in a lysis buffer (20 mM Tris pH 8.0, 10 mM EDTA, 5 mM BME, 0.1 mM PMSF) and lysed with a M-110L CF microfluidizer (Microrofluidics, MA). The pellet from a high-speed spin (100,000 g, 1 h) was resuspended in the lysis buffer and spun at a low speed (10,000 g, 20 min) to separate heavier inclusion bodies and other cell debris, followed by a second high speed spin. The crude membrane fraction was resuspended in a salt wash buffer (20 mM Tris pH 8.0, 0.5 M NaCl, 10 mM EDTA, 5 mM BME, 0.1 mM PMSF) and spun down at a high speed (100,000 g, 1 h). The pellet (the washed membrane fraction) was resuspended in a storage buffer (20 mM Tris pH 8.0, 100 mM NaCl, 20% Glycerol, 5 mM BME, 0.1 mM PMSF), topped with argon and frozen at -80°C.

### Protein extraction from cell membrane

Aliquots of QseC membrane fraction were mixed 1:1 with extraction buffer (20 mM Tris-HCl pH 7.6, 200 mM NaCl, 1 mM MgCl_2_, 1 mM BME, and 1 tablet of protease inhibitor cocktail per 10 ml) containing an extraction detergent at the various concentrations listed in Table 1 and incubated overnight at 6°C. The detergent-solubilized fraction was separated by centrifugation at 125,000 g for 2 h. The extraction efficiency was estimated by SDS-PAGE.

### Detergent exchange

The FC14-solubilized fraction of Etk was separated by centrifugation at 125,000 g for 2 h and diluted to the final FC14 concentration of 5 mM. Three columns with 5 ml of Ni-NTA agarose (Qiagen, CA) were pre-equilibrated in 0.5 mM FC14 and 20 ml of the FC14 solubilized Etk sample was loaded to each column. The first column was washed 5 times with 10 ml of 0.5 mM DDM; the second column was initially washed 5 times with 10 ml of 5 mM DDM and then 3 times with 10 ml of 0.5 mM DDM; the third column was initially washed 5 times with 10 ml of 15 mM DM and then 5 times with 10 ml of 8 mM DM. All "flow-through" and wash fractions were collected. The protein was then eluted 3 times with 5 ml of 250 mM imidazole in 0.5 mM DDM (1^st ^and 2^nd ^column) or 8 mM DM (3^rd ^Column) buffers.

### NMR spectroscopy and concentration measurements

A Varian 500 MHz NMR spectrometer equipped with automatic sample changer was used for NMR analysis. 130 *μ*l of 5.0 mM 2,2-dimethyl-2-silapentane-5-sulfonic acid (DSS) solution in D_2_O were added to 520 *μ*l of each sample. As a result, all NMR samples contained DSS at 1.0 mM, while concentrations of other sample components were decreased 1.25 times. All NMR spectra were acquired with 128 scans, 2.7s acquisition time and 1.5 s relaxation delay at 25°C using standard Varian 1D-^1^H-experiment with gradient water suppression. The sensitivity of this experiment is high enough to quantify the amount of buffer components at concentrations higher than 0.05 mM with a reasonable error < 5% (see Table 3). The (CH_3_)_3_-Si-group signal of 1.0 mM DSS was used both as the ^1^H chemical shift (0.0 ppm) and as the concentration reference. To calculate concentrations of the buffer components, the integrals of the signature detergent signals (Table 2) and of other buffer component signals were scaled to the integral of the DSS signal. The signal from the aliphatic chain of detergents, which partially overlapped with the methyl and/or methylene signals of hydrophobic residues, was only used for crosschecking concentration measurements.

In order to determine the detergent retention ratio, the concentrations of the detergent were measured both in the extraction buffer and in the PDC fraction. The concentrations of both the extraction and destination detergents were determined at various steps of the detergent exchange process: in the flow-through, wash, and elution fractions from the Ni-NTA column, as well as in the wash and elution buffers. The detergent concentration factor (*cf*) was defined as:

cf=fc−ic(n−1)⋅ic,
 MathType@MTEF@5@5@+=feaafiart1ev1aaatCvAUfKttLearuWrP9MDH5MBPbIqV92AaeXatLxBI9gBaebbnrfifHhDYfgasaacPC6xNi=xI8qiVKYPFjYdHaVhbbf9v8qqaqFr0xc9vqFj0dXdbba91qpepeI8k8fiI+fsY=rqGqVepae9pg0db9vqaiVgFr0xfr=xfr=xc9adbaqaaeGacaGaaiaabeqaaeqabiWaaaGcbaGaem4yamMaemOzayMaeyypa0tcfa4aaSaaaeaacqWGMbGzcqWGJbWycqGHsislcqWGPbqAcqWGJbWyaeaacqGGOaakcqWGUbGBcqGHsislcqaIXaqmcqGGPaqkcqGHflY1cqWGPbqAcqWGJbWyaaGccqGGSaalaaa@4176@

where *n *is the fold concentration, *ic *and *fc *are the initial and final detergent concentrations calculated from the NMR spectra of starting and concentrated samples.

### Velocity measurements by analytical ultracentrifugation

The analytical ultracentrifugation experiments were performed on the Beckman Optima XL-I (Beckman, CA). EnvZ, Etk, and Etk-NM overexpressed in *E. coli *were extracted by 18 mM FC14 for Etk, 18 mM FC14 or 20 mM DPh for EnvZ, and 18 mM FC12 for Etk-NM. The extraction detergent for EnvZ was exchanged with 0.5 mM DDM and 0.5 mM FC14, for Etk it was exchanged with 0.5 mM DDM and 2 mM FC12, and for Etk-NM it was exchanged with 2 mM FC12. Proteins were concentrated using the Vivaspin 50 kDa concentrators. The samples were then diluted to 0.8 OD using 50 mM NaCl, 20 mM Tris, pH 7.5, and the above mentioned concentration of the appropriate destination detergent. The density of each buffer was measured using a DMA 55 densitometer (Anton Paar, Austria).

Two samples of Etk-NM in FC12 were prepared. For one of them the density was adjusted with D_2_O to 1.0260 g/cm^3 ^to match the density of the detergent and the other was left unadjusted with the buffer density of 1.0105 g/cm^3^. One hundred fifty absorbance scans at 40,000 rpm and 20°C were collected for each sample using an AN60 rotor. The data were analyzed using ultrascan software [23]. Absorbance scans of all samples were first analyzed using the van Holde-Weischet method [20]. Absorbance scans of EnvZ in DDM exchanged from Dph and of Etk-NM in FC12 were fit by a finite element analysis. A one-component model was used to fit scans of EnvZ in DDM with the variance of 5.2 × 10^-4 ^and the number of runs equals to 16% and to fit scans of both adjusted and unadjusted Etk-NM in FC12 with the variance and the number of runs equal to 8.7 × 10^-5 ^and 13 %, and 5.1 × 10^-5 ^and 17%, respectively. The obtained buoyant mass for EnvZ of 29.7 kDa (fitted mass multiplied by (1 - *ρ*·v¯p
 MathType@MTEF@5@5@+=feaafiart1ev1aaatCvAUfKttLearuWrP9MDH5MBPbIqV92AaeXatLxBI9gBaebbnrfifHhDYfgasaacPC6xNi=xH8viVGI8Gi=hEeeu0xXdbba9frFj0xb9qqpG0dXdb9aspeI8k8fiI+fsY=rqGqVepae9pg0db9vqaiVgFr0xfr=xfr=xc9adbaqaaeGacaGaaiaabeqaaeqabiWaaaGcbaGafmODayNbaebadaWgaaWcbaGaemiCaahabeaaaaa@2EF5@), where *ρ *= 1.0013 g/cm^3 ^and v¯p
 MathType@MTEF@5@5@+=feaafiart1ev1aaatCvAUfKttLearuWrP9MDH5MBPbIqV92AaeXatLxBI9gBaebbnrfifHhDYfgasaacPC6xNi=xH8viVGI8Gi=hEeeu0xXdbba9frFj0xb9qqpG0dXdb9aspeI8k8fiI+fsY=rqGqVepae9pg0db9vqaiVgFr0xfr=xfr=xc9adbaqaaeGacaGaaiaabeqaaeqabiWaaaGcbaGafmODayNbaebadaWgaaWcbaGaemiCaahabeaaaaa@2EF5@ = 0.7345 cm^3^/g) was used to calculate Mp/Md for the monomer and the dimer using the method described in Step 4. Since the difference between the densities of adjusted and unadjusted samples of Etk-NM in FC12 was only 0.0155 g/cm^3^, we assumed that the fitted masses for both samples correspond to the molecular weight of the protein component of PDC only, because the density of the buffer matches that of the detergent.

## Abbreviations

IMP, Integral membrane protein; PDC, Protein-detergent complex; HT, High-throughput; Brij-35, polyethylene glycol(23)monododecyl ether; C12E8, dodecyl octaethylene glycol ether; *β*-OG, n-octyl-*β*-D-glucopyranoside; *β*-NG, n-nonyl-*β*-D-glucopyranoside; DM, n-decyl-*β*-D-maltopyranoside; DDM, n-dodecyl-*β*-D-maltopyranoside; FC12, DPC, n-dodecylphosphocholine; FC14, n-tetradecylphosphocholine; TX-100 (Also Triton X-100), *α*- [4-(1,1,3,3-tetramethylbutyl)phenyl]-*ω*-hydroxy-poly(oxy-1,2-ethanediyl)]; LDAO, lauryldimethylamine-N-oxide; Zw3.12, n-dodecyl-N, N-dimethyl-3-ammonio-1-propanesulfonate; Zw3.14, n-tetradecyl-N, N-dimethyl-3-ammonio-1-propanesulfonate; SDS, sodium dodecyl sulfate; DPh, disodium-N-lauryl-*β*-iminodipropionate.

## Authors' contributions

IM and WK conceived the study, conducted the NMR and ultracentrifuge analyses, conducted subsequent data analyses, participated in protein expressions, and drafted the manuscript. GK and CJ conducted protein expressions and assays. RR and SC participated in the NMR experimental design and coordination of the study and drafted the manuscript. All authors read and approved the final manuscript.
